# P3HT:Bebq_2_-Based Photovoltaic Device Enhances Differentiation of hiPSC-Derived Retinal Ganglion Cells

**DOI:** 10.3390/ijms20112661

**Published:** 2019-05-30

**Authors:** Chih-Chen Hsu, Yi-Ying Lin, Tien-Chun Yang, Aliaksandr A. Yarmishyn, Tzu-Wei Lin, Yuh-Lih Chang, De-Kuang Hwang, Chien-Ying Wang, Yung-Yang Liu, Wen-Liang Lo, Chi-Hsien Peng, Shih-Jen Chen, Yi-Ping Yang

**Affiliations:** 1 Department of Ophthalmology, Taipei Veterans General Hospital, Taipei 11217, Taiwan; chihchienym@gmail.com (C.-C.H.); yarmishyn@gmail.com (A.A.Y.); backyard0826@gmail.com (T.-W.L.); sjchen96@gmail.com (S.-J.C.); 2 Institute of Clinical Medicine, National Yang-Ming University, Taipei 11221, Taiwan; yyliu@vghtpe.gov.tw; 3 Institute of Pharmacology, National Yang-Ming University, Taipei 11221, Taiwan; s19609005@gm.ym.edu.tw (Y.-Y.L.); ylchang@vghtpe.gov.tw (Y.-L.C.); 4 Department of Medical Research, Taipei Veterans General Hospital, Taipei 11217, Taiwan; a8434040@hotmail.com; 5 School of Medicine, National Yang-Ming University, Taipei 11221, Taiwan; wangcy@vghtpe.gov.tw; 6 Department of Pharmacology, Taipei Veterans General Hospital, Taipei 11217, Taiwan; 7 Department of Ophthalmology, Taichung Veterans General Hospital, Taichung 40705, Taiwan; m95gbk@hotmail.com; 8 Department of Public Health and Institute of Public Health, National Yang-Ming University, Taipei 11217, Taiwan; 9 Department of Critical Care Medicine, Taipei Veterans General Hospital, Taipei 11217, Taiwan; 10 Division of Trauma, Department of Emergency Medicine, Taipei Veterans General Hospital, Taipei 11217, Taiwan; 11 Department of Chest, Taipei Veterans General Hospital, Taipei 11217, Taiwan; 12 Institute of Oral Biology, National Yang-Ming University, Taipei 11211, Taiwan; wllo@vghtpe.gov.tw; 13 Division of Oral and Maxillofacial Surgery, Department of Stomatology, Taipei Veterans General Hospital, Taipei 11217, Taiwan; 14 Department of Dentistry, School of Dentistry, National Yang-Ming University, Taipei 11211, Taiwan; 15 Department of Ophthalmology, Shin Kong Wu Ho-Su Memorial Hospital & Fu-Jen Catholic University, Taipei 11101, Taiwan; chpeng1008@gmail.com; 16 School of Pharmaceutical Sciences, National Yang-Ming University, Taipei 11211, Taiwan

**Keywords:** induced pluripotent stem cells (iPSC), retinal ganglia cells (RGC), poly-3-hexylthiophene (P3HT), photovoltaic

## Abstract

Electric field stimulation is known to affect various cellular processes, including cell fate specification and differentiation, particularly towards neuronal lineages. This makes it a promising therapeutic strategy to stimulate regeneration of neuronal tissues. Retinal ganglion cells (RGCs) is a type of neural cells of the retina responsible for transduction of visual signals from the retina to the brain cortex, and is often degenerated in various blindness-causing retinal diseases. The organic photovoltaic materials such as poly-3-hexylthiophene (P3HT) can generate electric current upon illumination with light of the visible spectrum, and possesses several advantageous properties, including light weight, flexibility and high biocompatibility, which makes them a highly promising tool for electric stimulation of cells in vitro and in vivo. In this study, we tested the ability to generate photocurrent by several formulations of blend (bulk heterojunction) of P3HT (which is electron donor material) with several electron acceptor materials, including Alq3 and bis(10-hydroxybenzo[h]quinolinato)beryllium (Bebq2). We found that the photovoltaic device based on bulk heterojunction of P3HT with Bebq2 could generate photocurrent when illuminated by both green laser and visible spectrum light. We tested the growth and differentiation capacity of human induced pluripotent stem cells (hiPSC)-derived RGCs when grown in interface with such photostimulated device, and found that they were significantly increased. The application of P3HT:Bebq2-formulation of photovoltaic device has a great potential for developments in retinal transplantation, nerve repair and tissue engineering approaches of treatment of retinal degeneration.

## 1. Introduction

It is generally known that endogenous electric fields play important roles in cell physiology and in controlling various important biological processes such as embryonic development, wound healing, tissue repair, cell growth and differentiation [1,2]. Therefore, stimulation of cells and tissues by exogenous electric fields offers great potential for modulation of these processes in clinical application. For example, the positive effect of application of electric field on neuronal regeneration has been known for decades [3,4], and recently found its way into clinical trials to treat spinal cord-injured patients [5,6].

The electric fields are known to orchestrate various processes of cell activity and physiology, including cell fate specification, stem cells differentiation and commitment during development [2]. Several studies have shown that electrical stimulation biases differentiation of stem cells towards neuronal lineages. Yamada et al. have shown that electrical stimulation of embryonic stem cells bias their differentiation towards a variety of neuron types, including motor neurons, interneurons, or their precursors [7]. Electrical activity also plays an important role in early retinal development. Tandon et al. reported that electrical stimulation drove retinal progenitor cells to differentiate into functional excitability and mature neuron [8].

Retina is the complex tissue, consisting of several types of specialized neural cells, including photoreceptors, responsible for converting energy of light photons into membrane electrical signals, conveyed by retinal ganglion cells (RGCs) into the brain cortex to form visual perception. Several eye diseases that lead to blindness are caused by degeneration of retinal cells, such as glaucoma, associated with degeneration of retinal ganglion cells, and retinitis pigmentosa, associated with degeneration of photoreceptors. The principle of electric stimulation has been applied for correction of pathologies associated with degeneration of photoreceptor cells, by placing electrode-based prosthetic devices into the retina to stimulate intact RGCs [9]. One of the promising approaches in retinal prosthesis technology is via the application of photovoltaic materials that are able to convey the energy of light into electric current to stimulate the adjacent neurons. For example, the silicon photodiode-based pixel photovoltaic device has been demonstrated to restore light sensitivity in rats with photoreceptor degeneration [10,11].

The organic semiconducting materials represent a promising alternative to the silicon-based photodiodes for the design of retinal photovoltaic devices, as they are characterized by higher flexibility, biocompatibility, lighter weight, cheaper cost and simplicity of production [12,13]. Poly(3-hexylthiophene) (P3HT) is one of the most promising organic photovoltaic materials for interfacing retinal neurons, as it possesses a number of valuable properties, such as lack of cytotoxicity and stability of its optoelectronic properties in the biological environment [14]. P3HT is a conjugated polymer that serves as an electron donor, and performs the best in generating photocurrent when combined with electron acceptor materials in bulk heterojunction (BHJ) architecture [15]. Moreover, in the study by Ghezzi et al., it was shown that even a single-component P3HT film was able to stimulate blind rat retinas [16].

In this study, we evaluated the availability of P3HT-based solar cell-like devices upon photoelectric stimulation to promote differentiation of retinal progenitors into RGCs. We tested various photovoltaic devices with distinct modifications to generate photocurrent, and among all modified photovoltaic devices, the device with the bulk heterojunction architecture containing electron donor P3HT and electron acceptor Bebq2 was shown to be the most efficient device to generate photocurrent. The P3HT:Bebq2 solar cell-like device provided an ideal interface for the cultivation of hiPSC-derived retinal progenitor cells and further enhanced their differentiation efficiency into RGCs.

## 2. Results

### 2.1. Development and Characterization of High Efficiency Solar Cell-Like Polymeric Materials

**Figure 1:**
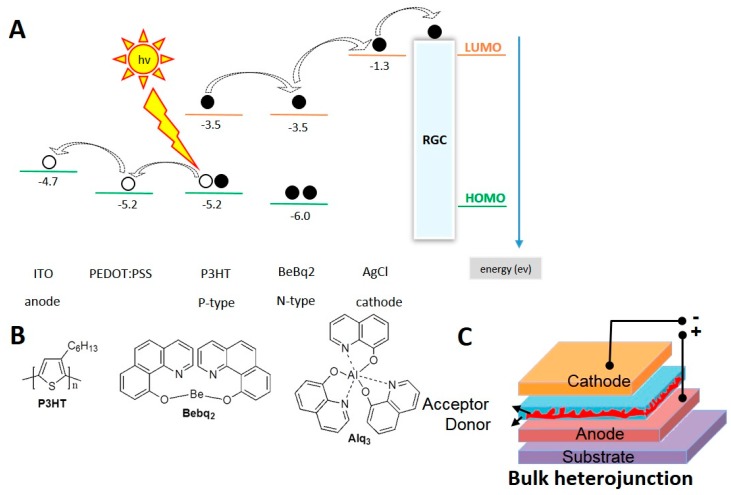
Schematic representation of high efficiency solar cell-like polymeric materials design; (**A**) poly(3-hexylthiophene) (P3HT) presented the same level of electronic energy level with BeBq2; (**B**) the formula structure of P3HT, bis(10-hydroxybenzo[h]quinolinato)beryllium (BeBq2), and tris-(8-hydroxyquinoline)aluminum (Alq3); (**C**) the internal structure of the solar-cell like polymeric material composed of both donor and acceptor layers arranged in bulk heterojunction.

Solar cell-like devices have been applied to establish a photoelectric field environment that is suitable for RGC growth and can promote differentiation [16,17]. Several optoelectronic materials, including P3HT, N719 dye, N3 dye, Alq3, BeBq2, Ir(ppy)3, Bphen, BCP and F4-TCNQ, have been shown as the candidates of the solar cell-like materials. The photovoltaics mechanism of the electron donor-acceptor mechanism is currently the foundation of the main compositions of high efficiency organic solar cells. The scheme in Figure 1A depicted the working principle of the solar cell. The photovoltaics mechanism shares an advantage of the bulk heterojunction architecture to widen the interface between donor and acceptor, maximizes the probability of charge pair separation and limits the charge recombination process. In this study, we developed a solar-cell like material with the main electron donor-acceptor composed of P3HT, Alq3 and BeBq2, as demonstrated in Figure 1B. In the composition of the solar cells, the interfacial layers namely a hole transport layer and an electron transport layer are inserted between the anode-photoactive and cathode-photoactive interfaces, respectively to improve the performance and stability of the bulk heterojunctions. The performance of bulk heterojunction devices is comparable with the layered architecture solar cells; besides, they also exhibit relatively higher environmental stability. The typical device architecture for normal and inverted solar cells is illustrated in Figure 1C.

Green laser has been proved that it can stimulate photovoltaic devices [18]. Therefore, we developed a multi-absorption device for blue, green and red color with the wavelength in the range of 450–475 nm, 495–570 nm and 620–750 nm, respectively. Herein, we also focused on the development of new materials for the device, which are capable to be excited by the above light sources. The surface of the solar cell-like device being seeded with RGC cells of which are anticipated to result in charge transportation. The series connection with blue, green and red sensors cultured upon RGC cells to form a solar cell-like device in the plate of patch clamp.

### 2.2. Development of a Three-Dimensional (3D) Culture System in the Purpose of Investigating iPSCs-Induced RGC Cells in an Accurate Representation of the In Vivo Environment and Study the Cultured RGC Cells on Multi-Color Absorption Solar Cell-Like Device

As it is known, mammalian retinas are composed of three neuronal layers, including retinal ganglion cells (RGCs). RGCs located in the vitreous side of the retinal layer, are called ganglion cell layer, and extend long axons from RGCs soma to various corresponding areas of lateral geniculate body. Numerous crucial data regarding RGC development and physiology have been obtained using the organotypic culture method or dissociated culture system [19]. However, considering the species difference resulting in the morphological and development variation, and ethics issues when acquiring ex vivo sources of human retina, development and characterization of RGCs from in vitro stem cells culture have been largely investigated. In a recent study, RGCs differentiation protocol, such as retinal serum-free floating culture of embryoid body-like aggregates with a quick reaggregation (SFEBq) culture has been proposed [20]. SFEBq provide three-dimensional self-organizing optic vesicle-like structures (3D retinal structure) with layered retinal neurons. The RGCs differentiation procedure was confirmed with RGC markers, including Brn3a, and/or Brn3b, which are responsible for dendritic stratification, axonal projections, and maintenance.

Hydrogel, a three-dimensional (3D) culture system, is capable of delivering higher degrees of control and organization of cellular environments, physiologically correlated cell migration, and provoke the formation of extensive 3D networks of cell-cell interactions. We differentiate human iPSCs into RGCs by the 3D retinal layer protocol mentioned above. Moreover, in order to safely enhance the generation efficiency of iPSC-differentiated specific retinal neurons and further shorten the differentiation time to well-functional retinal cells, effective growth factors and molecules were applied during the in vitro differentiation process.

The series connection with blue, green and red sensors cultured upon 3D-cultured RGC cells to form a solar cell-like device in the plate of patch clamp.

**Figure 2:**
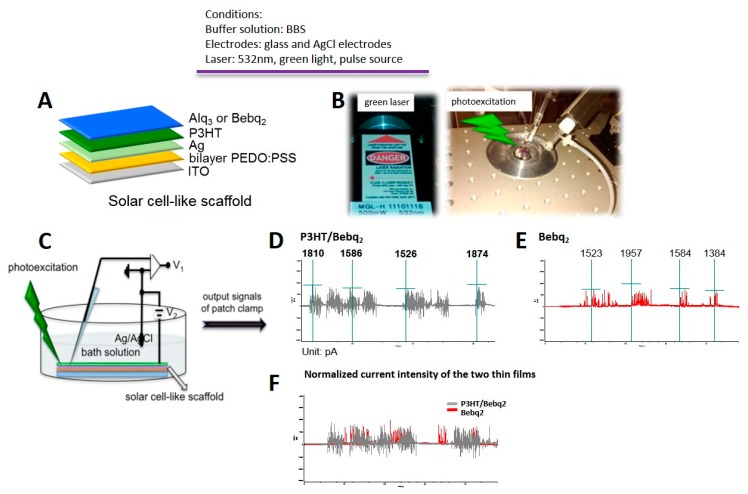
The procedures of detective platform for solar cell-like scaffold of patch clamp and the results of the current patch clamp. (**A**) The layered structure of this innovative solar cell-like photovoltaic device, composed of P3HT and Alq3. (**B**) the instrumental setup of the green laser utilized to stimulate the P3HT photovoltaic device. The incident green laser was 532 nm in wavelength from pulse source. The photovoltaic device was immersed in BBS, in which glass and AgCl electrodes was used to detect electric current when the incident green laser irradiated on the photovoltaic device. (**C**) scheme of measurement of photocurrent transmitted from the solar cell-like scaffold soaked in Ag/AgCl bath solution when irradiated under green laser light. The output current was further measured and recorded with patch clamp. (**D**–**F**) Intensity of the current from photovoltaic device. The photocurrent was both measured in P3HT/Bebq2 and Bebq2 only group, while P3HT/Bebq2 showed more light generated and shorted responsive time than Bebq2 only.

In order to confirm whether the solar cell-like device transfers the electrons/holes to form an optoelectronic circuit after being stimulated by light resource, we established the detective platform as the following Figure 2. The solar cell-like plate is prepared by spin coating procedures as Figure 2A, placed the light sensor plate into a dish filled with borate buffered saline (BBS) and irradiated the plate by green laser with the wavelength located at 532 nm, Figure 2B. An illustration of the patch clamp detection is presented in Figure 2C. Bi-electrical system, glass and AgCl electrodes, were used in the study. The results are shown in Figure 2D–F, Figure 2D,E showed the intensity of the current patch clamp coated within P3HT/Bebq2 and Bebq2 on the top surface of the plate, respectively. Under the normalized intensity condition of Figure 2F, the plate with P3HT/Bebq2 behaved shorter responsive time and triggered more light-generated electron/hole pairs than that of Bebq2 only. This consequence may reveal that while coating one more N-type optoelectronic material, a thin film upon the top of P3HT thin film would expand the interface of PN junction and engendered a strong photoconductive effect.

### 2.3. Differentiation of hiPSCs into Retinal Ganglion Cells (RGCs)

**Figure 3:**
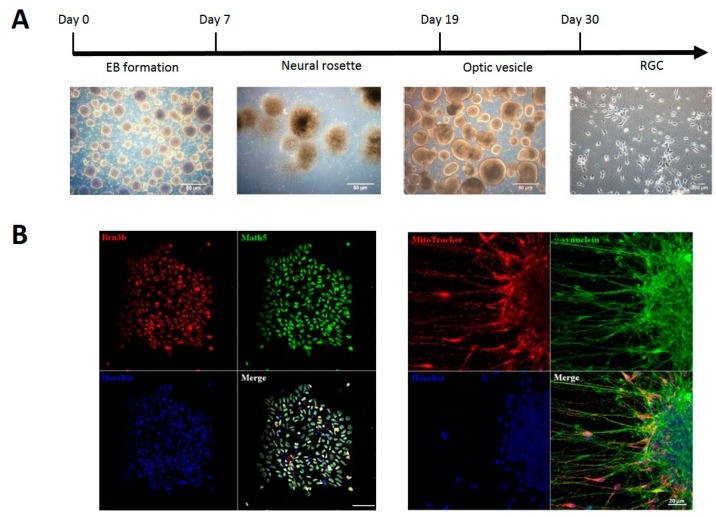
Differentiation of human induced pluripotent stem cells (hiPSCs) into retinal ganglion cells (RGCs). (**A**) Schematic outline of the process of differentiation of hiPSCs into RGCs; (**B**) expression of RGC positive markers detected by immunofluorescence microscopy Nuclei stained with 4′,6-diamidino-2-phenylindole (DAPI) and mitochondria with MitoTracker.

In this study, we used hiPSCs derived from peripheral blood mononuclear cells. These hiPSCs were characterized by alkaline phosphatase activity, expression of pluripotency markers and embryonic stem cell-like morphology with high nucleus to cytoplasm proportion, circular morphology and clear cell-to-cell boundaries (data not shown). The hiPSCs displayed typical undifferentiated hESC-like colony morphology. In the typical differential induction of the neural progenitor from ESCs in vitro, the neural rosette formation is observed, representative for the neural tube in vivo. Fundamentally, the neural tube and neural rosette were both characterized of apical-basal polarity, composing a pseudostratified layer of neuroepithelial cells. In the neural rosette, the inner surface presents the apical side while the outer surface presents the basal side. Additionally, in both the neural rosette and neural tube, the proliferating cells layered at the apical side, and thus, the differentiated cells clustered at the basal side. The neural rosettes derived from the evaginated and continuous neuroectodermal epithelium during retinogenesis in the SFEBq culture. For differentiation of hiPSCs into RGCs, we used the method adapted from Ohlemacher et al. [19,21]. First, embryoid bodies (EBs) were generated by day seven by cultivating hiPSCs in a suspension culture, which were further differentiated into neural rosettes and optic vesicles (OVs) by day 19 (Figure 3A). The neural rosettes could only normally have developed under correct apical-basal polarity. By day 30, RGCs with characteristic neural morphology were differentiated from Ovs (Figure 3A). These RGCs were characterized by expression of specific RGC nuclear markers BRN3B and Atoh7, as well as γ-synuclein and abundant mitochondria distributed in the axons. Due to the fact that Atoh7 are temporarily expressed during the process of retinogenesis, the expression of Atoh7 declined after RGCs are maturely differentiated from iPSC. Thus, we established RGCs from human iPSCs and confirmed the Atoh7 expression as one of the biomarker during RGCs differentiation, while we monitored γ-synuclein in mature RGCs. The class IV POU domain transcription factor Brn3b, is one of the earliest and crucial RGCs markers, which is downstream of Ath5. Brn3b was involved in the formation of RGCs, which have been proved in Math5 and Brn3b double null mice, RGCs presents a defect and loss during development [22]. The Brn3b expression was observed during our differentiation, indicating genetic and physiologic functionality (Figure 3B).

### 2.4. Design and Testing of Solar Cell-Like Device

**Figure 4:**
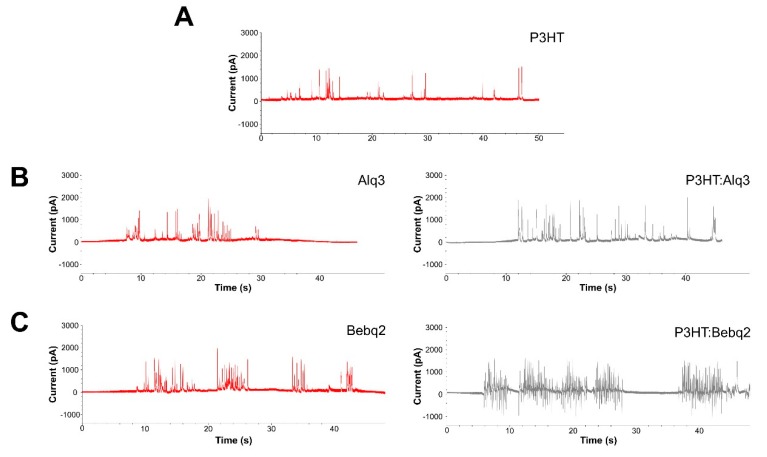
Photovoltaic efficiency analysis of solar cell-like devices. Electric activity of different formulations of photovoltaic materials upon illumination with a green laser at a wavelength of 532 nm: (**A**) P3HT only; (**B**) Alq3 only (left) and P3HT:Alq3 bulk heterojunction (right); (**C**) Bis(10-hydroxybenzo[h]quinolinato)beryllium (Bebq2) only (left) and P3HT:Bebq2 bulk heterojunction (right).

For a design of the active photo-sensor bulk heterojunction layer of a solar cell-like device, we used P3HT as an electron donor material, Alq3 and Bebq2 as electron acceptor materials (Figure 1A). Thus, the complete design of the device included the outer interface bulk heterojunction layer of P3HT:Alq3 or P3HT:Bebq2, followed by the layers of silver (Ag), poly(3,4-ethylenedioxythiophene) polystyrene sulfonate (PEDOT:PSS), and indium tin oxide (ITO) (Figure 1C). In order to identify whether this device was able to generate photocurrent when being stimulated under light exposure, it was demonstrated that the detection platform was established with a specific wavelength excitation, whereby the device was placed in a culture dish containing phosphate buffered saline (PBS), irradiated with a green laser at a wavelength of 532 nm and the current was detected by patch clamp. The control devices, in which the interface layer was composed of PEDOT:PSS and a single component of P3HT generated low frequency photocurrent (Figure 4A), so did the devices with the interface layers composed of Alq3 and Bebq2 only (Figure 4B,C, left). The highest photocurrent spike frequency was generated by a P3HT:Bebq2 bulk heterojunction-based device (Figure 4C, right), which was noticeably higher than that detected on a P3HT/Alq3-based device. The stimulation scheme of the solar cell-like device upon which is being seeded with RGC cells. Therefore, for further experiments we used a P3HT:Bebq2 bulk heterojunction-based design of photovoltaic device.

### 2.5. White Light Induces Photocurrent in P3HT:Bebq2 Solar Cell-Like Device

**Figure 5:**
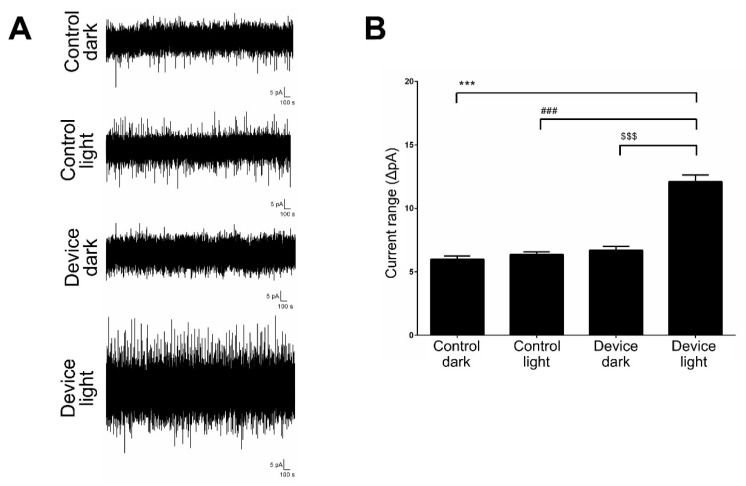
White light induces photocurrent in a P3HT:Bebq2 solar cell-like device. (**A**) Measurement of current induced in the P3HT:Bebq2 solar cell-like device by white light. Glass was used as a control; (**B**) quantification of white light-induced current. Mean values from xx independent measurements are shown with standard deviation error bars. *** *p* < 0.001, ### *p* < 0.01, $$$ *p* < 0.05 Student’s t-test, compared to control.

Next, after comparing the certain composition of photovoltaic materials and finding the proper combination of P3HT and BeBq2, it was necessary to identify whether an exterior white light source could stimulate the P3HT:Bebq2 solar cell-like device to generate photocurrent for neural cell stimulation. Therefore, we tested the P3HT:Bebq2 solar cell-like device under applied white light exposure, and recorded the electric current with the patch clamp pipette. The electric currents were recorded by the voltage clamp from the P3HT:Bebq2 surface of the device and glass control in the dark and after exposure to white light. The current amplitude range was significantly increased when measured on a P3HT:Bebq2 surface of the device as compared to the controls (Figure 5).

### 2.6. P3HT:Bebq2 Solar Cell-Like Device Stimulates Differentiation of Retinal Progenitors into RGCs

To investigate the effect of a P3HT:Bebq2-generated photocurrent on differentiation, retinal progenitor cells from the optic vesicles were seeded on top ofa P3HT:Bebq2 surface and on top of glass as a control and grown under illumination with white light. Ten days later, cells were immunostained for the specific RGC nuclear marker Atoh7 and axonal marker TUJ1. These RGCs differentiated from the optic vesicle were immunoreactive to TUJ1 marker. TUJ1 was a neuronal class III β-tubulin, expressing in microtubules derived from brain and RGCs, but not in retinal glial cells. RGCs presented TUJ1 immunoreactivity, which was observed in the cytoplasm of the soma, axons and dendrites.

**Figure 6:**
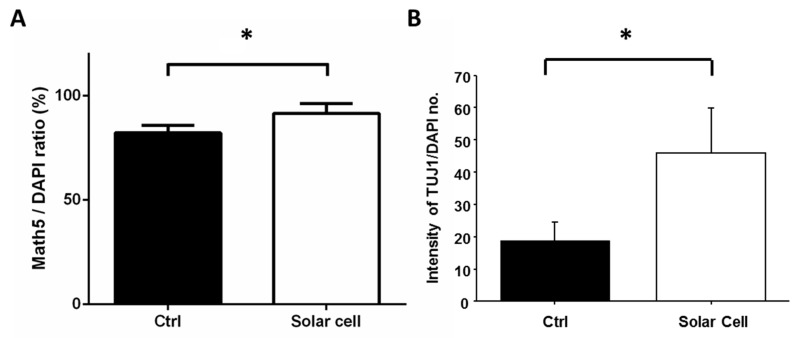
P3HT:Bebq2 solar cell-like device stimulates differentiation of retinal progenitors into RGCs. (**A**) Quantification of Math5 to DAPI ratio of RGCs grown on P3HT:Bebq2 surface; (**B**) intensity quantification of TUJ1 to DAPI number of RGCs grown on the surface of P3HT:Bebq2 solar cell-like device as compared to glass control. Mean values from 3 independent measurements are shown with standard deviation error bars. * *p* < 0.05, Student’s t-test.

RGCs grown on the P3HT:Bebq2 surface exhibited much denser neurite outgrowth morphology as compared to the control (Figure 6A). The ratio of cells expressing Math5 and TUJ1 to the total number of nuclei stained with DAPI was higher among RGCs grown on top of the solar cell as compared to the control (Figure 6B), thus indicating more efficient differentiation towards RGC lineage.

## 3. Discussion

Recently, modulation of various cellular activities by interfacing with organic photovoltaic materials is finding a growing biomedical application. The thiophene-based materials such as P3HT are the most thoroughly investigated and widely used, as they absorb light in most of the visible spectrum, are highly biocompatible, flexible, simple in production [12,13]. P3HT is a photoactive conjugated polymer with semiconductor properties that serves as an electron donor material, whereas its combination with electron acceptor materials results in charge separation and generation of the current. The intimate blend of electron donor and acceptor phases, known as bulk heterojunction architecture, has proven to be highly effective in the generation of photocurrent and electric stimulation of interfaced cells [12,13,14,16]. In our study, we tested two types of bulk heterojunction architecture of P3HT-based solar cell devices, P3HT:Alq3 and P3HT:Bebq2, based on Alq3 and Bebq2 electron acceptor materials, respectively. We found that upon illumination with green laser (532 nm), P3HT:Bebq2 could generate a high frequency photocurrent unlike P3HT:Alq3 (Figure 3). Moreover, we showed that the P3HT:Bebq2-based device could generate photocurrent upon illumination with the natural light of the visible spectrum (Figure 4).

Electric cues play important role in regulating cell physiology [2]. P3HT-based interfaces have proven to be effective in stimulating various physiological activities of both electrogenic and non-electrogenic cells. In a pioneering study, the P3HT:PCBM bulk heterojunction-based device was shown to be able to stimulate primary neurons in a light-dependent manner [14]. In addition to modulating the activity of electrogenic cells, electrical stimulation is known to affect various kinds of activities of non-electrogenic cells, including proliferation, differentiation, cell polarity, and therefore, finding growing application for tissue regeneration. The P3HT-based photovoltaic device has been shown to stimulate neural differentiation and neurite outgrowth of PC12 cells [23]. In a study by Yang et al., it was shown that the photoactive P3HT nanoweb was able to enhance neurogenesis of human stem cells [24]. Similarly, in our study, we show that the P3HT:Bebq2-based device has a positive effect on differentiation of iPSC-derived RGCs and enhances neurite outgrowth. Given that a number of retinal diseases are associated with degeneration of RGCs, we believe that the P3HT:Bebq2 formulation of photovoltaic device has a great potential for the developments in retinal transplantation, nerve repair and tissue engineering approaches of treatment of retinal degeneration.

Whereas in our study we investigated the planar interface between the P3HT-based solar cell-like device and RGCs, other design strategies can also be applied for specific purposes. One promising approach to stimulate cells is by miniaturization of the P3HT-based photovoltaics in the form of nanoparticles. Such approach is particularly promising for application in vivo, as it would not require complicated surgical procedures as in the case of planar interface-based devices. For example, P3HT-based nanoparticles have been applied to modulate calcium ion flux when internalized by HEK-293 cells [25,26]. Similarly, P3HT nanoparticles were successfully used to photostimulate Hydra polyps, thus demonstrating successful application in vivo [25]. In the study by Yang et al., P3HT was applied in the form of nanofibrils to efficiently stimulate the neural differentiation of human stem cells [24]. This approach significantly enhances the surface area of P3HT, and may provide topographical cues for neurite outgrowth. Therefore, we believe our P3HT:Bebq2-based device can be investigated to be applied in the form of nanoparticles or nanofibrils to stimulate the growth and maintenance of RGCs in vivo.

## 4. Materials and Methods

### 4.1. Differentiation of hiPSCs into OVs

hiPSCs were differentiated into optic vesicles (OVs) as previously described [21]. hiPSCs were dissociated from the culture dish using Versene (Gibco, Waltham, MA, USA) and cultured in suspension with a 3:1 mixture of mTeSR1 (STEMCELL Technologies, Inc. Cambridge, MA, USA) and a Neural Induction Medium (NIM) consisting of DMEM/F12 (1:1), N2 supplement, MEM non-essential amino acid (NEAA; Gibco, Waltham, MA, USA) and heparin (Sigma-Aldrich, St. Louis, Mo, USA). The medium was changed to 1:1 mTeSR1/NIM on day two, 1:3 mTeSR1/NIM on day four, and to 100% NIM on day six. On day seven, embryoid bodies (EBs) were seeded into a 6-well plate containing NIM supplemented with 10% FBS (Gibco, Waltham, MA, USA) to promote the attachment of EBs within the next two days. The medium was then changed every 2–3 days until day 16 of differentiation. By day 16, neural rosettes (NRs) were formed, which were further cultivated in a retinal differentiation medium (RDM: DMEM/F12 (3:1)) supplemented with 2% B-27 (without vitamin A, Invitrogen), 1X NEAA (Gibco, Waltham, MA, USA) and 1% penicillin/streptomycin (Gibco, Waltham, MA, USA). On day 19, the NRs were mechanically separated from the adherent cultures, so as to keep the rosettes intact and retain surrounding colony cells. The NRs were grown in a RDM suspension culture until they developed into three-dimensional OV-like structures.

### 4.2. Differentiation of OVs into RGCs

The OVs were stimulated to differentiate into RGCs by culturing for one day in DMEM/F12 containing 5% FBS (Gibco, Waltham, MA, USA), N-2 supplement (Gibco, Waltham, MA, USA), and DAPT (STEMCELL Technologies, Inc. Cambridge, MA, USA). On the following day, the medium was replaced with DMEM/F12 containing N-2 supplement (Gibco, Waltham, MA, USA), and 10 μM DAPT (STEMCELL Technologies, Inc. Cambridge, MA, USA). On day three, the medium was replaced with a Neurobasal Medium (Gibco, Waltham, MA, USA) containing a B-27 supplement (Gibco, Waltham, MA, USA), DAPT (STEMCELL Technologies, Inc. Cambridge, MA, USA), and L-Glutamine (Gibco, Waltham, MA, USA). In the later culture, the cells were cultivated in a Neurobasal Medium containing B-27 and L-Glutamine. In order to isolate single RGCs, TrypLE Express (Invitrogen, Waltham, MA, USA) was used to break up the OVs into single cell suspensions. The cells suspension was centrifuged (1000 rpm, 5 min) and the supernatant was removed while cells were seeded onto poly-D-Lysine (PDL)/Laminin (Thermo Scientific, Waltham, MA, USA)-coated coverslips. Coverslips were placed in RDM and the medium was changed every two days until nerve growth was complete.

### 4.3. Immunofluorescence Staining

Live cells were cultured in MitoTracker Red (Cell Signaling, Danvers, MA, USA) at 37 °C for 30 min and then with 4% paraformaldehyde (Sigma-Aldrich, St. Louis, MO, USA) at room temperature for 30 min. Next, cells were incubated with blocking buffer containing 10% goat serum and 0.3% Triton X-100 in PBS for 1 h and then incubated with primary antibodies against Brn3b (1:250; Santa Cruz, Dallas, TX, USA), Math5 (1:500; Millipore, Bethesda, MA, USA), and Thy1 (1:500; Abcam, Cambridge, UK) at 4 °C overnight. The cells were then washed with PBS and incubated with secondary antibodies diluted in a blocking buffer containing Hoechst (1:10,000; Thermo Scientific, Waltham, MA, USA) for 1 h at room temperature. The following secondary antibodies include sheep and Alexa 488 goat anti-rabbit IgG, Alexa 594 goat anti-rabbit IgG, Alexa 488 goat anti-mouse IgG (Thermo Scientific, Waltham, MA, USA), and Alexa 594 goat anti-mouse IgG (Thermo Scientific, Waltham, MA, USA). Fluorescent staining of mitochondria is then performed using the MitoTracker Red CMXRos (Cell Signaling, Danvers, MA, USA). Finally, the cells were detected by confocal microscopy (Zeiss LSM 700, Zeiss, Jena, Germany).

### 4.4. Preparation of the Solar Cell-Like Device

Different organic polymer optoelectronic materials were sequentially coated on indium tin oxide (ITO) glass. The first conductive layer of poly(3,4-ethylenedioxythiophene) doped with polystyrene sulfonate (PEDOT:PSS) was spin-coated onto ITO substrate (side length 18 mm; thickness 180 μm; resistance 10 Ω/sq), using the following spinning parameters: The first step—2000 rpm, rotation duration 30 s; the second step—5000 rpm, rotation duration 40 s, followed by heating to 160 °C for 3–8 min. Ag, the second layer, was deposited by the first spinning at 2000 rpm for 30 s and the second spinning at 5000 rpm for 40 s, followed by heating to 120 °C for 5 min. The last layer of P3HT, P3HT:Bebq2 or P3HT:Alq3 was deposited by spinning at 2000 rpm for 45 s, heating to 180 °C for 2 min, followed by 120 °C for 2 min. This procedure was repeated three times. After all procedures, the surface was modified and coated by PDL/Laminin, as described previously [27].

### 4.5. Photostimulation

A high power light-emitting diode (peak at 532 nm, LED) was used to create circular illumination spots for light stimulation. The photosensing device was placed into a dish filled with phosphate buffered saline (PBS) and irradiated by green LED. The patch clamp was used to detect the current changes in the device. Photocurrent measurements were performed at room temperature in PBS with patch pipettes (4–6 MΩ) filled with the same solution, using the MultiClamp 700B amplifier (Molecular Devices) in a voltage-clamp mode. The response was amplified (low-pass filtered at 1kHz, digitized at 20kHz) and the data were analyzed with pCLAMP10.

### 4.6. Statistical Analysis

Statistical significance was assessed by an unpaired Student’s t-test for parametric data and a *p*-value less than 0.05 was defined as statistically significant.

## 5. Conclusions

We believe that the P3HT:Bebq2-formulation of the photovoltaic device has a great potential for developments in retinal transplantation, nerve repair and tissue engineering approaches of treatment of retinal degeneration.

## Abbreviations

RGCs	Retinal ganglion cells
hiPSC	Human induced pluripotent stem cells
EBs	Embryoid bodies
OVs	Optic vesicles
NRs	Neural rosettes
P3HT	Poly(3-hexylthiophene)
Alq3	Tris-(8-hydroxyquinoline)aluminum
BeBq2	Bis(10-hydroxybenzo[h]quinolinato)beryllium
PCBM	Phenyl-C61-butyric acid methyl ester
PEDOT:PSS	poly(3,4-ethylenedioxythiophene) polystyrene sulfonate
ITO	Indium tin oxide
BH	Bulk heterojunction
PBS	Phosphate buffered saline
SFEBq	Serum-free floating culture of embryoid body-like aggregate with quick reggregation
DAPI	4′,6-diamidino-2-phenylindole
FBS	Fetal bovine serum
RDM	Retinal differentiation medium
NIM	Neural Induction Medium
DMEM/F12	Dulbecco’s Modified Eagle Medium: Nutrient Mixture F-12
NEAA	Non-Essential Amino Acids
PDL	poly-D-Lysine
GAPDH	Glyceraldehyde-3-Phosphate Dehydrogenase
LED	Light-emitting diode
